# Analysis of common and coding variants with cardiovascular disease in the diabetes heart study

**DOI:** 10.1186/1475-2840-13-77

**Published:** 2014-04-12

**Authors:** Jeremy N Adams, Laura M Raffield, Barry I Freedman, Carl D Langefeld, Maggie CY Ng, J Jeffrey Carr, Amanda J Cox, Donald W Bowden

**Affiliations:** 1 Program in Molecular Genetics and Genomics, Wake Forest School of Medicine, Winston-Salem, NC 27157, USA; 2 Center for Genomics and Personalized Medicine Research, Wake Forest School of Medicine, Winston-Salem, NC 27157, USA; 3 Center for Diabetes Research, Wake Forest School of Medicine, Winston-Salem, NC 27157, USA; 4 Department of Internal Medicine – Nephrology, Wake Forest School of Medicine, Winston-Salem, NC 27157, USA; 5 Division of Public Health Sciences, Department of Biostatistical Sciences, Wake Forest School of Medicine, Winston-Salem, NC 27157, USA; 6 Department of Radiologic Sciences, Wake Forest School of Medicine, Winston-Salem, NC 27157, USA; 7 Department of Biochemistry, Wake Forest School of Medicine, Winston-Salem, NC 27157, USA

**Keywords:** Coronary artery calcified plaque, Type 2 diabetes mellitus, Cardiovascular disease, Genetic risk score

## Abstract

**Background:**

Type 2 diabetes mellitus (T2DM) is a major cardiovascular disease (CVD) risk factor. Identification of genetic risk factors for CVD is important to understand disease risk. Two recent genome-wide association study (GWAS) meta-analyses in the Cohorts for Heart and Aging Research in Genomic Epidemiology (CHARGE) consortium detected CVD-associated loci.

**Methods:**

Variants identified in CHARGE were tested for association with CVD phenotypes, including vascular calcification, and conventional CVD risk factors, in the Diabetes Heart Study (DHS) (n = 1208; >80% T2DM affected). This included 36 genotyped or imputed single nucleotide polymorphisms (SNPs) from DHS GWAS data. 28 coding SNPs from 14 top CHARGE genes were also identified from exome sequencing resources and genotyped, along with 209 coding variants from the Illumina HumanExome BeadChip genotype data in the DHS were also tested. Genetic risk scores (GRS) were calculated to evaluate the association of combinations of variants with CVD measures.

**Results:**

After correction for multiple comparisons, none of the CHARGE SNPs were associated with vascular calcification (p < 0.0014). Multiple SNPs showed nominal significance with calcification, including rs599839 (*PSRC1*, p = 0.008), rs646776 (*CELSR2*, p = 0.01), and rs17398575 (*PIK3CG*, p = 0.009). Additional *COL4A2* and *CXCL12* SNPs were nominally associated with all-cause or CVD-cause mortality. Three SNPs were significantly or nominally associated with serum lipids: rs3135506 (Ser19Trp, *APOA5*) with triglycerides (TG) (p = 5×10^−5^), LDL (p = 0.00070), and nominally with high density lipoprotein (HDL) (p = 0.0054); rs651821 (5′UTR, *APOA5*) with increased TGs (p = 0.0008); rs13832449 (splice donor, *APOC3*) associated with decreased TGs (p = 0.0015). Rs45456595 (*CDKN2A*, Gly63Arg), rs5128 (*APOC3*, 3′UTR), and rs72650673 (*SH2B3*, Glu400Lys) were nominally associated with history of CVD, subclinical CVD, or CVD risk factors (p < 0.010). From the exome chip, rs3750103 (*CHN2,* His204Arg/His68Arg*)* with carotid intima-medial thickness (IMT) (p = 3.9×10^−5^), and rs61937878 (*HAL,* Val549Met*)* with infra-renal abdominal aorta CP (AACP) (p = 7.1×10^−5^). The unweighted GRS containing coronary artery calcified plaque (CAC) SNPs was nominally associated with history of prior CVD (p = 0.033; OR = 1.09). The weighted GRS containing SNPs was associated with CAC and myocardial infarction (MI) was associated with history of MI (p = 0.026; OR = 1.15).

**Conclusions:**

Genetic risk factors for subclinical CVD in the general population (CHARGE) were modestly associated with T2DM-related risk factors and CVD outcomes in the DHS.

## Introduction

Type 2 diabetes mellitus (T2DM) is a major public health concern throughout the world. T2DM risk factors include gender, family history, smoking, abnormal lipid metabolism, and genetics [1]. T2DM is associated with several comorbidities, including retinopathy, nephropathy, and especially cardiovascular disease (CVD) [2].

CVD is a major complication of T2DM. An individual’s risk for CVD is two to four times greater if they have T2DM relative to an individual not affected by T2DM [3,4]. In 2007, heart disease was noted on the death certificates of 68% of individuals with diabetes [2,5]. Diabetes is an independent CVD risk factor, with relative risk for CVD-associated mortality 2.1 for men and 4.9 for women compared to those not affected by T2DM [3,4]. There is increasing evidence that both genetic and environmental factors contribute to this risk.

CVD risk can be assessed in many ways. A widely used measure of subclinical CVD is coronary artery calcified plaque (CAC) assessed by computed tomography (CT) imaging. CAC is a powerful predictor of all-cause and CVD-related mortality [5-7]. Calcified atherosclerotic plaque (CP) can also be measured in the carotid artery (CarCP) and infra-renal abdominal aorta (AACP). Another widely used measure is thickness of the intima media layer of the carotid arteries (IMT) [7]. Importantly, CAC and IMT appear to capture different aspects of CVD [6].

Identification of heritable risk factors for CVD and other complications of T2DM are important to improve our understanding of an individual’s risk. An extensive genetic analysis of CVD in the general population was recently performed in the Cohorts for Heart and Aging Research in Genomic Epidemiology (CHARGE) consortium [8,9]. These studies consisted of genome wide association study (GWAS) meta-analyses identifying loci associated with CAC, myocardial infarction (MI), and IMT. These studies identified multiple CVD associated loci. However, less than 10% of the CHARGE samples were affected by T2DM. In the present study, the relevance of the CHARGE results were tested in a T2DM-enriched sample from the Diabetes Heart Study (DHS), where more than 80% of subjects had T2DM [5,7].

## Methods

### Subjects

The DHS evaluates the genetic and epidemiological causes of CVD in individuals with T2DM. Ascertainment, recruitment, and examination have been described in detail [7]. Briefly, siblings with T2DM and without advanced nephropathy were recruited, with unaffected siblings also recruited when possible. T2DM was defined as diabetes developing after 35 years of age, with initial treatment using a combination of, exercise and/or oral agents, not solely insulin, and in the absence of historical evidence of ketoacidosis. Diabetes diagnosis was confirmed upon entrance to the study by measurement of fasting glucose and glycated hemoglobin (HbA_1C_) testing. The 1,208 European American individuals included in this analysis were from 473 families.

### Clinical evaluation

Study protocols were approved by the Institutional Review Board at Wake Forest School of Medicine. Participants provided written informed consent prior to participation. Examinations were conducted in the General Clinical Research Center of the Wake Forest Baptist Medical Center and included interviews for medical history and health behaviors, anthropometric measures, resting blood pressure, electrocardiography, fasting blood sampling for laboratory analyses (blood lipid profile, fasting glucose, HbA_1C_, and high-sensitivity C-reactive protein (CRP)) and spot urine collection. Individuals were considered hypertensive if they were prescribed anti-hypertensive medication or had blood pressure measurements exceeding 140 mmHg (systolic) or 90 mmHg (diastolic).

CAC, CarCP, and AACP were measured using fast-gated helical CT scanning, and calcium scores were computed as previously described and reported as Agatston scores [10,11]. IMT was measured by high-resolution B-mode ultrasonography with a 7.5-MHz transducer and a Biosound Esaote (AU5) ultrasound machine (Biosound Esaote, Inc., Indianapolis, IN) as previously described [12]. Not all measurements were available in all participants.

Vital status was determined from the National Social Security Death Index maintained by the United States Social Security Administration. For those participants confirmed as deceased, length of follow-up was determined from date of the initial study visit to date of death [6,13]. For deceased participants, copies of death certificates were obtained from relevant county Vital Records Offices to confirm cause of death. For all other participants the length of follow-up was determined from the date of the initial study visit to the December 31, 2012. Causes of death were categorized based on information contained in death certificates as CVD-related (myocardial infarction, congestive heart failure, cardiac arrhythmia, sudden cardiac death, peripheral vascular disease, and stroke) or either cancer, infection, end-stage renal disease, accidental, or other (including obstructive pulmonary disease, pulmonary fibrosis, liver failure and Alzheimer’s dementia).

### DHS GWAS and imputed data

Genomic DNA was purified from whole-blood samples obtained from subjects using the PUREGENE DNA isolation kit (Gentra Systems., Minneapolis, MN). DNA was quantitated using standardized fluorometric readings on a Hoefer DyNA Quant 200 fluorometer (Hoefer Pharmacia Biotech, Inc., San Francisco, CA). Each sample was diluted to a final concentration of 5 ng/μL.

A GWAS was completed using the Affymetrix Genome-wide Human SNP Array 5.0 (Affymetrix, CA, USA) as reported [14]. Genotype calling was completed using the BRLLM-P algorithm in Genotyping Console v4.0 (Affymetrix). Samples failing to meet an intensity quality control (QC) threshold and those failing to meet a minimum acceptable call rate of 95% were excluded from further analyses (n = 7). An additional 39 samples were included as blind duplicates within the genotyping set to serve as QC samples; the concordance rate for these blind duplicates was 99.0 ± 0.72% (mean ± standard deviation (SD)). Exploratory analyses of genotype data were performed using PLINK v1.07 (http://pngu.mgh.harvard.edu/purcell/plink/) and samples with poor quality genotype calls, gender errors, or unclear/unexpected sibling relationships were excluded from further analysis. Exclusion criteria for single nucleotide polymorphism (SNP) performance included call rate <95% (n = 11,085), Hardy-Weinberg Equilibrium (HWE) p-value <1×10^−6^ (n = 332), and minor allele frequency (MAF) <0.01 (n = 57,382); 371,951 SNPs were retained for analysis.

Additional genotype data were obtained by imputation from the GWAS. Imputation of 1,000 Genomes Project SNPs was completed using the program IMPUTE2 (http://mathgen.stats.ox.ac.uk/impute/impute_v2.html) and the Phase I v2, cosmopolitan (integrated) reference panel, build 37 [15]. SNPs that were used for imputation were required to have low missingness and show no significant departure from HWE expectations. To maximize the quality of imputation, the samples were not pre-phased. Only imputed SNPs with a confidence score >0.90 and information score >0.50 were used.

### Individual SNP genotyping

For those genes implicated in the CHARGE meta-analyses as potential risk loci, exonic variants contained in the National Heart Lung and Blood Institute Grand Opportunity Exome Sequencing Project (NHLBI GO-ESP). Coding SNPs identified as possibly damaging or probably damaging by PolyPhen2 [16] with a minor allele frequency (MAF) less than 0.2 were selected for genotyping. Fourty-four single nucleotide polymorphisms (SNPs) from 17 genes were genotyped in the DHS. Genotyping was performed using the Sequenom Mass ARRAY genotyping system (Sequenom, San Diego, CA) and PCR primers were designed using the Mass ARRAY Assay Design 3.4 Software (Sequenom). An additional 41 quality control (QC) samples were included in the genotyping analysis to serve as blind duplicates. The concordance rate for the blind duplicates was 100%. For all SNPs the minimum acceptable call frequency was 95%. The average call frequency was 97.3 ± 0.009% (mean ± SD). Samples with genotyping efficiency rates <90% were excluded from further analysis. Twenty-eight SNPs from 14 genes were carried forward to analysis after QC. Genotyped SNPs are listed in Additional file 1.

### Exome chip

Additional SNPs for those genes implicated in the CHARGE meta-analyses as potential risk loci were also identified as captured by the Illumina® HumanExome BeadChips (Illumina® Inc., San Diego, CA) for which genotype data was available in the DHS. For DHS Exome Chip data, genotype calling was completed using Genome Studio Software v1.9.4 (Illumina). Samples failing to meet a minimum acceptable call rate of 98% (n = 3) were excluded from further analyses. An additional 58 samples were included as blind duplicates within the genotyping set to serve as QC samples; the concordance rate for blind duplicates was 99.9 ± 0.0001% (mean ± SD). Exclusion criteria for SNP performance included call rate <99% (n = 972), monomorphic SNPs (n = 157,754) and Hardy-Weinberg Equilibrium p-value <1×10^−6^ (n = 26); 88,483 SNPs were retained for analysis. Additional QC of Exome Chip data set was completed to exclude samples with poor quality genotype calls, gender errors, or unclear/unexpected sibling relationships.

### Genetic risk scores

Genetic risk scores (GRS) were calculated as previously described [17]. Both unweighted GRS and GRS weighted by SNP effect size were derived for two sets of SNPs previously reported to be associated with CAC or CAC and MI. SNPs included in both GRS are shown in Additional file 2. One set of 12 SNPs had documented effects on CAC (Score 1; 1a = unweighted, 1b = weighted). We created a second GRS from 8 SNPs associated with CAC and MI (Score 2; 2a = unweighted, 2b = weighted). Unweighted scores were derived by adding the number of effect alleles for each SNP for each person. The SNPs were also weighted by their previously reported effect sizes [8,9]. For the weighted scores, the number of effect alleles possessed by an individual at a particular SNP locus was multiplied by a weight derived from that SNP’s effect size contribution to the total effect size for all SNPs included in the GRS. For individuals missing genotype data for a particular SNP, the mean genotype calculated in the DHS for that given SNP was assigned [18]. For all GRS, the effect allele was assigned as the allele associated with an increase in CAC or increased risk for CAC.

All derived GRS (1a, 1b, 2a, and 2b) were tested for association with CAC, CarCP, AACP, IMT, prior history of CVD events, prior history of MI, all-cause mortality, and CVD-cause mortality to evaluate whether the GRS were a measure of genetic contributions to either clinical or subclinical CVD.

### Statistical analysis

Allele frequencies were calculated for a sub-set of unrelated individuals and departure from HWE was calculated from a group of unrelated samples using a chi-squared goodness-of-fit test implemented in PLINK v1.07. Association between the SNP genotypes and CVD measures was examined using variance component methods computed using SOLAR v4.3.1 (Texas Biomedical Research Institute, San Antonio, Tx, USA) which accounted for family structure. Each trait was examined using additive, dominant, and recessive models of inheritance. Most of the associations were observed under the additive model, however, there were associations seen under only the dominant or recessive models. Continuous variables were transformed prior to analysis to approximate conditional normality. Age, gender, T2DM-affection status, and body mass index (BMI) were used as covariates in all single variant association analyses. Additional covariates (e.g. cholesterol medication use, T2DM duration, and smoking) were also tested, but did not meaningfully impact the results. Statistical significance for all single SNP analysis was calculated using the Li & Ji method to determine the effective number of SNPs using SOLAR v. 4.3.1. Statistical significance was set at p < 2.16×10^−4^. Power calculations for dichotomous traits were run using CaTS (University of Michigan School of Public Health http://www.sph.umich.edu/csg/abecasis/CaTS/). Power calculations for continuous traits were run using Quanto (University of Southern California http://hydra.usc.edu/gxe/).

GRS were considered as continuous variables. Relationships between the GRS and CAC, CarCP, AACP, history of CVD, and history of MI were examined using marginal models with incorporation of generalized estimating equations. These models use a sandwich estimator of the variance under exchangeable correlation in order to account for familial correlation [17]. Relationships between GRS and both all-cause and CVD-cause mortality were examined using Cox proportional hazards models with sandwich-based variance estimation due to the inclusion of related individuals. Associations were adjusted for age, gender, BMI, smoking status (current or prior smoking), hypertension, cholesterol medications, and prior CVD as indicated. All analyses were performed in SAS 9.3 (SAS Institute, Cary, NC) and statistical significance was accepted at p < 0.05.

## Results

Characteristics of the DHS sample are summarized in Table 1. The mean age of the sample was 61.5 years at examination. 1013 (83.86%) were T2DM-affected, the mean BMI approached 32 kg/m^2^, and slightly more than 50% (643) were female. The characteristics of the DHS samples are broadly representative of T2DM-affected patients in the general population: older, relatively obese, and with significant risk factors and history of CVD.

**Table 1:** Demographic characteristics of DHS samples (data shown are mean ± SD, unless specified otherwise)

	**Total**
Number	1208
Age	61.5 ± 9.35
Female (%)	643 (53.2)
BMI (kg/m^2^)	31.8 ± 6.49
T2DM Affected (%)	1013 (83.9)
Diabetes duration (years)	10.41 ± 7.1
Metabolic syndrome (%)	1029 (85.2)
Lipid medication (%)	540 (44.7)
CAC	1662.5 ± 3160.7
CarCP	312.9 ± 672.1
AACP	10949.3 ± 15748.8
IMT (mm)	0.676 ± 0.134
Cholesterol (mg/dL)	186.8 ± 42.4
LDL (mg/dL)	105.1 ± 32.7
HDL (mg/dL)	43.1 ± 12.5
Triglycerides (mg/dL)	201.4 ± 132.1
History of CVD (%)	471 (38.99)
Deceased (%)	222 (18.38)
CVD Deceased (% of deceased)	100 (45.05)

We pursued multiple paths of analysis to assess the relevance of genetic loci implicated in various measures of CVD in the general population to another sample enriched for T2DM. Thus specific SNPs from the CHARGE subclinical CVD analysis [8,9] were tested *in silico* or directly genotyped to test for association in the DHS. In addition, the analysis has been expanded to include additional coding variants from the CHARGE loci risk and further expanded to assess the association of GRS, i.e. the cumulative effect of multiple associated SNPs, on CVD related traits. All analyses were performed in a subset of T2D-affected individuals alone and revealed similar results (data not shown).

### Testing CHARGE variants from DHS GWAS and imputation data

In an attempt to replicate the findings from the CHARGE studies, a total of 36 SNPs with available GWAS data were tested for association with clinical and subclinical CVD traits as well as CVD risk factors. No SNPs were significantly associated after correction for multiple comparisons (p < 0.0014), although several SNPs showed nominal association (Table 2). Among these, rs599839 near *PSRC1* (β = −0.31, p = 0.008) and rs646776 near *CELSR2* (β = −0.38, p = 0.01) were nominally associated with CAC, while rs17398575 near *PIK3CG* was associated with AACP (β = 11.0, p = 0.0054); and SNPs in *COL4A2* (rs3809346, rs4773144) and near *CXCL12* (rs1746048) were nominally associated with all-cause or CVD-cause mortality.

**Table 2:** Nominally significant results for the GWAS and imputation SNPs with measures of CVD and lipids

			**Additive**			**Dominant**			**Recessive**		
**SNP**	**Gene**	**Trait**	**P-value**	**β value**	**SE**	**P-value**	**β value**	**SE**	**P-value**	**β value**	**SE**
rs1746048	*CXCL12*	All-cause mortality	0.005	0.29	0.12	0.005	0.32	0.12	0.28	0.43	0.44
rs599839	*PSRC1*	CAC	0.008	−0.31	0.12	0.009	−0.36	0.14	0.27	−0.36	0.32
rs646776	*CELSR2, PSRC1, SORT1*	CAC	0.013	−0.31	0.12	0.010	−0.38	0.15	0.41	−0.28	0.34
rs17398575	*PIK3CG*	AACP	0.015	8.74	3.58	0.009	11.03	4.20	0.60	4.85	9.33
rs3809346	*COL4A1, COL4A2*	CVD-cause mortality	0.026	−0.18	0.08	0.36	−0.11	0.12	0.004	−0.39	0.14
rs4773144	*COL4A1, COL4A2*	CVD-cause mortality	0.040	−0.16	0.08	0.43	−0.095	0.12	0.007	−0.38	0.14

Associations were examined under additive, dominant and recessive genetic models. CAC = coronary artery calcified plaque; AACP = infrarenal abdominal aortic calcified plaque SE = standard error.

### Genotyped SNP results

To investigate the genes implicated by the CHARGE studies 28 exonic SNPs in 14 genes were identified in the NHLBI GO-ESP database, directly genotyped in the DHS and tested for association with clinical CVD, subclinical CVD, and CVD risk factors. The mean MAF for the genotyped SNPs were 0.0306 (0.0004-0.1617). The genotyped SNPs, along with gene, amino acid change, minor allele, and MAF can be found in Additional file 1.

Table 3 contains results from the association analysis with the genotyped SNPs. Three SNPs were significantly associated with CVD risk factors. rs3135506 (Ser19Trp) in *APOA5* was associated with increased triglyceride (TG) (p = 5×10^−5^), nominally associated with decreased low density lipoprotein (LDL)-cholesterol (p = 0.0007), and nominally associated with decreased high density lipoprotein (HDL)-cholesterol (p = 0.0066). rs651821 (5′ untranslated region (UTR)) also in *APOA5*, was nominally associated with increased TG concentration (p = 0.00080) while rs138326449 (splice donor) in *APOC3* was associated with decreased TG concentrations (p = 0.0015). Several other SNPs including rs45456595 (*CDKN2A*, Gly63Arg), rs5128 (*APOC3*, 3′UTR), and rs72650673 (*SH2B3*, Glu400Lys) were nominally associated (at p < 0.010) with history of CVD, subclinical CVD, or CVD risk factors.

**Table 3:** Association results for the genotyped exonic variants with clinical and subclinical CVD and blood lipids

		**Additive**			**Dominant**			**Recessive**		
**SNP**	**Trait**	**P-value**	**β value**	**SE**	**P-value**	**β value**	**SE**	**P-value**	**β value**	**SE**
rs3135506	Triglycerides	**5×10**^**−5**^	0.189	0.465	3×10^−4^	0.17	0.31	7×10^−4^	0.93	0.27
rs3135506	LDL	0.0012	−9.48	2.92	7×10^−4^	−10.1	2.99	0.65	10.1	22.4
rs3135506	HDL	0.0066	−0.20	0.07	0.011	−0.19	0.07	0.13	−0.63	0.42
rs651821	Triglycerides	8×10^−4^	0.15	0.04	0.004	0.14	0.05	0.005	0.5	0.18
rs651821	History of CVD	0.02	0.27	0.11	0.08	0.23	0.13	0.009	1.29	0.56
rs138326449	Triglycerides	0.0017	−0.97	0.31	0.0017	−0.97	0.31	NA	NA	NA
rs138326449	HDL	0.13	1.16	0.46	0.013	1.16	0.46	NA	NA	NA
rs45456595	IMT	0.0022	0.07	0.02	0.0022	0.07	0.02	NA	NA	NA
rs5128	Triglycerides	0.0096	0.10	0.04	0.03	0.09	0.04	0.01	0.41	0.16
rs72650673	Triglycerides	0.0084	0.77	0.29	0.0084	0.77	0.29	NA	NA	NA

Associations were examined under additive, dominant and recessive genetic models. Bold indicates statistical significance. History of CVD = prior reports of CVD events, IMT = carotid intima-media thickness, SE = standard error.

### Exome chip results

At total of 209 exonic SNPs with available genotype data from the exome chip were evaluated for association with clinical CVD, subclinical CVD, and CVD risk factors. Table 4 shows the significant results for the association of the SNPs from the Exome chip with subclinical and clinical CVD traits as well as CVD risk factors. One SNP rs3750103 (His204Arg/His68Arg) in *CHN2* was associated with IMT under the recessive model (β = 0.17, p = 3.9×10^−5^). A second SNP, rs61937878 (Val549Met) in *HAL* was significantly associated with AACP (β = 85.5, p = 7.1×10^−5^). Additional SNPs (rs61735307, *SERPINI1*; rs10496236, CTNNA2; rs11073922, *NGRN*) were nominally associated with various lipid measures IMT.

**Table 4:** Association results for SNPs from the DHS Exome Chip with measures of CVD and cholesterol

		**Additive**			**Dominant**			**Recessive**		
**SNP**	**Trait**	**P-value**	**β value**	**SE**	**P-value**	**β value**	**SE**	**P-value**	**β value**	**SE**
rs3750103	IMT	0.35	0.007	0.007	0.81	0.002	0.007	**3.9×10**^**−5**^	0.17	0.04
rs61937878	AACP	**7.1×10**^**−5**^	85.45	21.38	**7.1×10**^**−5**^	85.45	21.38	NA	NA	NA
rs61735307	IMT	2.6×10^−4^	0.24	0.06	2.6×10^−4^	0.24	0.06	NA	NA	NA
rs10496236	Cholesterol	3.2×10^−4^	−0.05	0.02	0.0032	0.0031	0.02	9.2×10^−4^	−0.16	0.05
rs11073922	IMT	0.21	0.007	0.005	0.71	0.002	0.006	7.2×10^−4^	0.06	0.019

Associations were examined under additive, dominant and recessive genetic models. Bold indicates statistical significance. AACP = infrarenal abdominal aortic calcified plaque, IMT = carotid intima-media thickness, SE = standard error.

### Genetic risk score

Finally, we determined the combined effect of the CHARGE SNPs on CVD traits. We analyzed 4 genetic risk scores containing different sets of SNPs and weighting method combinations for potential associations with CAC, CarCP, AACP, prior history of CVD, prior history of MI, and all-cause and CVD-cause mortality. The unweighted GRS containing CAC associated SNPs (GRS 1a) was associated with history of CVD events (p = 0.033; OR = 1.09). GRS 2b, the weighted risk score containing SNPs associated with both CAC and MI, was associated with history of MI (p = 0.026; OR = 1.15). No other associations were observed. Significant results for the GRS analysis are shown in Table 5.

**Table 5:** Significant association results for the GRS with CVD

**GRS**	**Trait**	**P-value**	**OR**	**95% CI**
1a	History of CVD	**0.033**	1.09	1.01 – 1.19
2b	History of MI	**0.026**	1.15	1.02 – 1.34

Bold indicates statistical significance. History of CVD = prior reports of CVD events, History of MI = prior reports of Myocardial Infarction, GRS = genetic risk score, 1a = unweighted risk score with CAC associated SNPs, 2b = weighted risk score with CAC and MI associated SNPs, OR = odds ratio, 95% CI = 95% confidence interval.

## Discussion

We assessed whether results seen in the CHARGE consortium GWAS meta-analyses were applicable to a T2DM enriched sample in the DHS. In addition, CHARGE analyses were extended by including coding variants in genes implicated in the CHARGE studies and testing GRS created from combinations of SNPs previously associated in CHARGE. We were unable to detect any associations that reached statistical significance in the initial analysis of CHARGE top-hit SNPs when applying a conservative Bonferoni correction; however several nominal associations were observed with clinical and subclinical CVD traits (Table 2).

A further investigation into the genes implicated in this study reveal a wide range of biological functions. Several genes with associated SNPs have some potential biological rationales. SNPs rs3809346 and rs4773144 located in the *COL4A2* gene were nominally associated with CVD mortality (Table 2). *COL4A2* encodes the protein collagen type IV alpha 2 which is the major component of basement membranes [19]. SNP rs17398575, near *PIK3CG*, was nominally associated with AACP. *PIK3CG* encodes for an enzyme that phosphorylates phosphoinositides. It is an important modulator of extracellular signals, including those elicited by E-cadherin-mediated cell-cell adhesion, with an important role in maintenance of the structural and functional integrity of epithelia [20]. Rs1746048, nominally associated with all-cause mortality (p-value of 0.005), is downstream of *CXCL12. CXCL12* encodes a stromal cell-derived alpha chemokine which can activate lymphocytes and may have a role in the cancer metastasis [21]. It is also a chemoattractant for T-lymphocytes and monocytes [22]. Activities of this protein and its receptor induce a rapid rise in the level of intracellular calcium ions and chemotaxis. Rs646776, downstream of *CELSR2,* was nominally associated with CAC. *CELSR2* encodes for a protein in the cadherin family that does not interact with catenins. It is thought that these proteins are involved in contact-mediated communication. However, the specific function has not been determined. *CELSR2* and *PSRC1* (rs599839) have been associated with both coronary artery disease and total cholesterol concentration [23].

We further investigated the genes implicated by the CHARGE studies by genotyping exonic SNPs and investigated further exonic SNPs from the DHS Exome chip. Exonic SNPs from CAC- and CVD-associated genes in CHARGE were found to be associated with lipid traits in DHS (Table 3). Additional coding variants were asociated with subclinical CVD traits and CVD risk factors (Table 4).

Several genes with known relevant biological functions were found in the investigation of coding SNPs. APOA5 (rs3135506, associated with TG concentrations and nominally with LDL concentrations; p-value = 5×10^−5^ and 7×10^−4^ respectively, and rs651821, nominally associated with triglyceride concentrations; p-value = 8×10^−4^; Table 3), which is a component of HDL, was shown associated with TG concentrations and coronary artery disease [24,25]. APOC3, a component of very low density lipoprotein (VLDL), is thought to delay catabolism of TG-rich particles and impact TG concentrations [26]. In DHS, *APOC3* was nominally associated with TG concentrations and HDL (rs138326449; p-value = 0.0017 and 0.013 respectively; rs5128 with TG; p-value = 0.0096) (Table 3). SNP rs3750103 in *CHN2* was associated with IMT with a p-value 3.9×10^−5^ (Table 4). *CHN2* encodes for the protein chimerin 2, which plays a role in the proliferation and migration of smooth muscle cells [27]. A second SNP, rs61937878, associated with AACP (p-value = 7.1×10^−5^; Table 4), is located in *HAL. HAL* encodes the protein histidine ammonia-lyase, which catalyzes the first step in histidine catabolism. Finally rs10496236 in *CTNNA2* was nominally associated with total cholesterol concentrations (p-value = 3.2×10^−4^; Table 4). *CTNNA2* encodes for catenin (cadherin-associated protein), alpha 2 which functions as a linker between cadherin adhesion receptors and the cytoskeleton to regulate cell-cell adhesion, predominantly in the central nervous system, and it has been associated with late onset Alzheimer’s disease [28].

Finally, we assessed whether GRS of CHARGE SNPs associated with CAC or CAC and MI provides a useful tool to predict CAC or CVD in the DHS. We found that GRS containing CAC risk SNPs or SNPs associated with increased CAC and MI were associated with CVD events in the DHS (Table 5). This indicates that CAC risk SNPs have potential to be used for identification of CVD risk in populations enriched for T2DM. The present studies were performed in European American subjects; results require replication in members of other population ancestries.

An important consideration in any study of this type is the statistical power of the study. This study has moderate power to identify associations with dichotomous trait and very high power to detect associations with continuous traits. For example, at a MAF of 0.35 we have 40% power to detect an association with history of CVD with an odds ratio of 1.15. For a quantitative trait example, this study has greater than 80% power to detect an association between a SNP with 0.11 MAF and a β-value of 0.1 for triglyceride levels.

## Conclusions

Taken together, these results provide evidence that the variants and genes implicated by the earlier CHARGE association studies affect clinical and subclinical CVD risk as well as CVD risk factors in European American individuals with T2DM. This study investigated known or suspected CVD associated variants and genes. However, these variants may not account for the individual’s full risk as there may be additional risk variants that are not covered by conventional techniques such as the *Haptoglobin* duplication [29]. Additional studies need to be performed to investigate the regions of the genome that aren’t easily covered by modern genotyping technology.

## Abbreviations

AACP: Infra-renal abdominal aortic calcified plaque; BMI: Body mass index; CAC: Coronary artery calcified plaque; CarCP: Carotid artery calcified plaque; CHARGE: Cohorts for Heart and Aging Research in Genomic Epidemiology; CRP: C-reactive protein; CVD: Cardiovascular disease; DHS: Diabetes Heart Study; GRS: Genetic risk score; GWAS: Genome wide association study; HbA1c: Glycated hemoglobin; HDL: High density lipoprotein; IMT: Intima media thickness; LDL: Low density lipoprotein; MAF: Minor allele frequency; MI: Myocardial infarction; NHLBI GO-ESP: National Heart Lung and Blood Institute Grand Opportunity Exome Sequencing Project; QC: Quality control; SNP: Single nucleotide polymorphism; SD: Standard deviation; TG: Triglycerides; T2DM: Type 2 diabetes mellitus; UTR: Untranslated region; VLDL: Very low density lipoprotein.

## Competing interests

The authors declare they have no competing interests.

## Authors’ contributions

JNA perfomed the SNP genotyping, calculated the GRS, performed all the statistical analysis, and wrote the manuscript; LMR assisted in the calculation of the GRS and reviewed the manuscript; BIF was involved in the conception of the DHS, participated in subject recruitment and clinical assessment, and reviewed the manuscript; CDL performed the GWAS imputation, contributed to statistical analyses, and reviewed the manuscript; MCYN assisted with the calculation of the GRS and reviewed the manuscript; JJC was involved in the conception of the DHS, participated in subject recruitment and clinical assessment, and reviewed the manuscript; AJC performed the clustering and QC for the DHS GWAS and Exome Chip, performed the GWAS and Exome association analyses, and assisted with the manuscript preparation; DWB designed and supervised the DHS, conceived the investigation, and assisted with the manuscript preparation. All authors read and approved the final manuscript.

## Supplementary Material

Additional file 1 **Genotyped SNPs carried forward to analysis for clinical and subclinical CVD as well as blood lipid traits.** MA %, Minor Allele %. Click here for file

Additional file 2 **List of SNPs used in the GRS.** GRS 1a and 1b contained SNPs associated with CAC. GRS 2a and 2b contained SNPs associated with CAC and MI. Click here for file
